# *E2f1* Overexpression Reduces Aging-Associated DNA Damage in Cultured Cerebral Endothelial Cells and Improves Cognitive Performance in Aged Mice

**DOI:** 10.1155/omcl/3242282

**Published:** 2025-07-28

**Authors:** Sheelu Monga, Samantha Flores, Maria Pilar Blasco-Conesa, Syed M. Rahman, Brian Noh, Pedram Peesh, Bhanu Priya Ganesh, Sean P. Marrelli, Louise D. McCullough, Jose Felix Moruno-Manchon

**Affiliations:** Department of Neurology, McGovern Medical School at the University of Texas Health Science Center at Houston, 6431 Fannin Street, Houston 77030, Texas, USA

**Keywords:** aging, cognition, DNA damage, E2F1, endothelial cells, itaconate

## Abstract

As we age, cerebral endothelial cells (CECs) are less efficient in maintaining genome integrity and accumulate DNA damage. DNA damage in the brain endothelium can lead to the impairment of the blood–brain barrier (BBB), which is a major factor in brain dysfunction and dementia. Thus, identifying factors that regulate DNA repair in the brain endothelium can prevent brain dysfunction associated with aging. E2F1 is a transcription factor that regulates the expression of genes associated with DNA repair, among other functions. We hypothesize that E2F1 is downregulated in the brain vasculature of mice with aging and that E2F1 upregulation can improve cognitive function. We found that in the brain endothelium, E2F1 was significantly less phosphorylated, which is associated with its transcriptional activity, in the brain vasculature of aged mice and cultured CEC derived from aged mice compared with those from young mice. We found that *E2f1* overexpression reduced DNA damage in cultured CEC, and targeting the brain vasculature to overexpress *E2f1* improved cognition and increased the expression of genes associated with BBB integrity in aged mice. From RNA sequencing data from cultured CEC, we found that *E2f1* overexpression significantly upregulated *Acod1*, which codes for aconitate decarboxylase-1 (ACOD1), an enzyme that produces itaconate. We also found that 4-octyl itaconate (4-OI), a derivative of itaconate, reduced DNA damage, promoted cell proliferation, and restored endothelial barrier function from oxidative stress in cultured CEC. Thus, our study identifies the E2F1-ACOD1 axis as a molecular pathway that can protect the brain endothelium from oxidative stress and aging.

## 1. Introduction

A healthy vascular endothelium is essential to supply oxygenated blood to brain cells and maintain low and selective permeability of the blood-brain barrier (BBB), and it is critical for the drainage of interstitial fluid [1]. However, with aging, brain vessels undergo functional and morphological changes, many of which are associated with different types of dementia, such as Alzheimer's disease (AD) and small vessel disease [1]. With aging, the endothelial cells of the brain vasculature exhibit profound changes that negatively affect cerebral blood flow, BBB permeability, and the architecture of the cerebral vasculature [2]. However, the molecular mechanisms and signaling pathways that are affected by aging and contribute to the impairment of the brain vasculature are not fully understood.

All cells in our body are exposed every day to multiple exogenous and endogenous factors that negatively affect the integrity of our genome, leading to DNA damage. Fortunately, all cells have DNA repair mechanisms that protect the genome from these insults. However, these mechanisms and the signaling pathways that regulate DNA repair become less efficient with aging, contributing to the accumulation of DNA damage, including in endothelial cells [3]. Enhanced DNA damage occurs in the brain vessels of the human cortex both with aging and in the early stages of the progression of AD pathology [4]. Persistent DNA damage in endothelial cells may lead to increased expression of genes associated with inflammation [5], reduction of cerebral blood flow [6], disruption of the BBB integrity [7, 8], neurovascular dysfunction, and enhanced neuroinflammation [9]. One of the most important stressors that induce DNA damage in the brain endothelium is oxidative stress, caused by free radicals that accumulate in the brain with aging [10]. Thus, identifying factors that regulate DNA repair pathways in the brain vasculature could contribute to developing therapeutic strategies to prevent disruption of the brain endothelium and mitigate the progression of cerebrovascular diseases associated with aging.

E2F1 is a member of the transcription factor E2F family. It participates in cell cycle control, DNA replication, DNA repair, DNA damage checkpoint control, apoptosis, autophagy, development, and differentiation [11–14]. Most of the biological functions of E2F1 are executed through its capacity to activate gene transcription. E2F1 promotes DNA repair and maintains genome integrity by binding to DNA damage sites and facilitating DNA acetylation, which changes chromatin to a more relaxed conformation, and hence different DNA repair proteins can access [15–20]. The requirement of E2F1 for the DNA repair process is evidenced by the fact that E2F1 deletion results in DNA repair inefficiency and DNA damage accumulation [17, 19, 21].

The roles of E2F1 in endothelial cells have been investigated in different tissues such as skin [22, 23], lungs [24], placenta [25], heart [26], and breast [27]. In the brain, E2F1 has been mostly implicated in the progression of glioblastoma [28–30]. Other functions of E2F1 in different brain cell types are the progression from proliferation to differentiation of oligodendrocytes [31] and neurogenesis [32, 33] but also can mediate neuronal death [34–36]. However, the study of its role in the brain vasculature has been limited thus far. Importantly, no studies have investigated the role of E2F1 in the brain vasculature with aging. Given that DNA repair pathways are downregulated with aging and that E2F1 maintains DNA integrity, we hypothesized that E2F1 is downregulated in the brain vasculature with aging.

In this study, we discovered that E2F1 was downregulated in the brains of aged mice compared with the brains of young mice, and its phosphorylated form, which indicates its transcriptional activity, was reduced in the brain microvessels and in cultured cerebral endothelial cell (CEC) derived from aged mice, compared with those from young mice. We found that the reduced phosphorylation of E2F1 was associated with enhanced DNA damage in the brain microvessels isolated from aged mice. Importantly, overexpressing *E2f1* in cultured CEC derived from aged mice reduced DNA damage. Delivering an adeno-associated virus (AAV) construct with high tropism for the brain vasculature (AAV-BR1) into aged mice to overexpress *E2f1* improved spatial memory and contextual learning, compared with control aged mice. We explored the possibility that the cognitive improvement caused by overexpressing *E2f1* in the brain vasculature of aged mice was likely due to an increased expression of genes associated with BBB permeability. Furthermore, analyzing the transcriptome of *E2f1*-overexpressing cultured CEC, we found that genes associated with immune response are the most highly upregulated compared with control CECs. Among these genes, we found that *Acod1*, which codes for aconitate carboxylase-1, an enzyme that synthesizes the immunoregulatory molecule itaconate, was significantly elevated. The treatment of cultured CEC from aged mice with a derivative of itaconate promoted cell proliferation, protected cells from DNA damage, and allowed cells to restore their endothelial barrier function after being exposed to oxidative stress.

## 2. Materials and Methods

### 2.1. Animals and Treatment

4-month-old (m/o) and 20-m/o C57BL/6J male and female mice were obtained from Jackson laboratories (#000664). Mice were housed in the installations of the Center for Laboratory Medicine and Care (CLAMC) at the University of Texas McGovern Medical School. Mice were maintained on a 12 h light/12 h dark schedule with constant temperature and humidity. Mice were fed with mouse lab pellets. Access to food and water was *ad libitum*. Investigators and CLAMC veterinarians warranted that those mice were not exposed to stress, pain, or injury.

To overexpress *E2f1* preferentially in the cerebral endothelium, 4-m/o and 20-m/o male mice (6–12 mice/age/vector) were injected with AAV (BR1)-CAG-mE2F1-T2A-GFP, or with AAV (BR1)-CAG-T2A-GFP as control via the carotid artery. The AAV capsid variant BR1 has a high tropism for the BBB-associated endothelium and allows durable expression (>660 days) [37]. These AAVs were designed and produced by SignaGen Laboratories. AAVs were diluted in saline solution and administered to mice at 5 × 10^10^ viral genomes per mouse (100 µL/mouse). Mice were maintained for 2 months until behavior testing was performed, followed by euthanasia.

### 2.2. Isolation and Culture of Primary Mouse CECs

We used the Adult Brain Dissociation Kit (MACS Miltenyi Biotec, #130-107-677) to isolate CEC from the brains of 4-m/o and 20-m/o old C57BL/6J male mice as previously described [38, 39]. Immediately after obtaining cell suspensions from each mouse, cells were plated in separate wells of 6-well plates previously coated with 0.1% porcine gelatin. The cells were plated with Complete Mouse Endothelial Cell Medium/w Kit (Cell Biologics, #M1168), which contains 5% FBS, 0.1% heparin, 0.1% hydrocortisone, 0.1% ECGS, 0.1% VEGF, 0.1% EGF, and 1% antibiotic–antimycotic solution. Endothelial cells are resistant to puromycin, and it was used at 4 µg/mL with the complete medium for 48 h to selectively maintain CEC in culture. After 48 h, the medium was replaced with fresh complete medium without puromycin. When cells reached 100% confluence (usually 1 week after cell isolation), cells were subcultured using 0.1% trypsin-EDTA and plated in 12-well plates, 96-well plates, or in inserts of 24-well transwell plates for the corresponding experiments.

For 4-octyl Itaconate (4-OI) treatments, we used 4-OI from Cayman Chemical Company (#25374). For hydrogen peroxide (H_2_O_2_) treatments, we used H_2_O_2_ from Millipore-Sigma (#1.08600). Cultured CECs from three 20-m/o male mice were pretreated with different concentrations of 4-OI (10, 25, and 50 µM) for 1 h. Then, the medium was aspirated, cells were washed once with PBS, and replaced with medium containing H_2_O_2_ (500 µM), or ultra-pure water as a vehicle in which cells were incubated overnight.

For transfections, CECs from four 20-m/o mice were cultured in 12-well plates at a density of 40,000 cells/well. 48 h after plating, cells were incubated with Opti-MEM (ThermoFisher Scientific, #31985062) and a mix of Lipofectamine 3000 transfection reagent (ThermoFisher Scientific, #L3000001) and DNA plasmid (0.4 µg/well) for 2 h. Then, the medium was replaced with basal medium, and cells were incubated for 72 h until subsequent treatments. The plasmids that we used were: pCMV-HA (Addgene, #32530), pCMV-HA-E2F1 (Addgene, #24225) [40], and pCAG-GFP (Addgene, #11150).

### 2.3. Isolation of Cerebral Microvessels From Adult Mice

Brains from 4-m/o and 20-m/o male mice (5–6 mice/age) were dissected, and the cortices were homogenized in MCDB 131 medium (ThermoFisher, #14190144) using a loose-fit 7-mL dounce tissue homogenizer and centrifuged (2000 *g*, 2 min, 4°C). After centrifugation, the pellets were resuspended in 15% (wt/v) dextran-DPBS and centrifuged (10,000 *g*, 15 min, 4°C). The pellets were again resuspended with DPBS and filtered through a 40 µm strainer. Microvessels remained in the filter and were fixed with 4% paraformaldehyde-DPBS for 10 min. The filter was inverted, and microvessels were collected in a 50 mL tube with MCDB medium and centrifuged. The pellets were resuspended in 200 µL PBS. 50 µL of microvessel suspensions were placed on a microscope slide and air-dried overnight for subsequent immunostaining [38, 39, 41].

### 2.4. Relative Gene Expression

We used the RNeasy Mini Kit (Qiagen, #74104) to isolate total RNA from cultured cells, and the iScript Reverse Transcription SuperMix (Bio-Rad, #1708840) to synthesize cDNA. We used the service of Qiagen for RNA sequencing analyses.

For qPCR, we combined cDNA with iTaq Universal SYBR® Green Supermix (Bio-Rad, #1725121). We used Bio-Rad CFX384 touch device to run qPCR reaction with these setting: 95°C for 3 min, and 40 cycles of 95°C for 10 s, and 55°C for 30 s.

Using RNA sequencing data, we established log10 *p* value > 2 to select the most significant differentially expressed genes, and log2 fold change <−1.5 and >1.5 to select the most downregulated and upregulated differentially expressed genes, respectively.

The relative expression of the genes of interest (*E2f1*, *Acod1*, *Plvap*, *Esam*, *Cldn1*, and *Jam2*) was calculated with the double delta Ct method relative to the expression of *Actb or Gapdh*. The sequences of the primers are in Table 1.

### 2.5. Western Blotting

Mouse brains from 4- and 20-m/o mice of both sexes (3–6 mice/sex/age) were homogenized with a dounce homogenize in RIPA buffer (150 mM NaCl, 1% Nonidet P40, 0.5% sodium deoxycholate, 0.1% SDS and 50 mM Tris/HCl pH 8.0), with a cocktail of phosphatase and protease inhibitors (Sigma Aldrich). Homogenate was lysed with 3 cycles of freezing-thawing (5 min in dry ice; 30 s in a water bath at 37°C; and 5 min in ice). Lysate was cleared by centrifugation at 15,000 × *g* for 15 min at 4°C, and the supernatant was collected. Protein concentration was analyzed by the Pierce Bicinchoninic Acid Protein Assay Kit (ThermoFisher Scientific, #23225). Samples were run in 8%–16% Mini-PROTEAN TGX gels (Bio-Rad), and then proteins were transferred onto a PVDF membrane. The membrane was blocked with 5% non-fat dry milk (1 h at RT), washed with TBS-Tween (0.1%), and incubated with a solution of primary antibody overnight at 4°C. We used anti-ACOD1 (Cell Signaling, #17805, 1:1,000) and anti-β-actin (Cell Signaling, #3700, 1:3000) as a housekeeping protein. The next day, the membrane was incubated with a solution of secondary antibody conjugated with HRP (1 h, RT). We used SuperSignal West Femto Maximum Sensitivity Substrate (ThermoFisher, #34094) to detect the chemiluminescent signal. The band intensity from each lane of the protein of interest was quantified with the ImageJ software and related to the band intensity of the corresponding lane of β-actin.

### 2.6. Immunostaining of Cultured CECs and Microvessel Fractions

Samples were fixed with a solution of 4% PFA/PBS for 10 min at RT. Then, permeabilized with 0.1% Triton-×100 for 10 min at RT. A solution of 5% bovine serum albumin in PBS was used as a blocking solution. Samples were blocked overnight at 4°C. Samples were then incubated with the primary antibodies overnight at 4°C. We used anti-γH2AX (Millipore, 05-636, 1:250), pE2F1 (Millipore, MABE1782, 1:250), and claudin-5 (Invitrogen, #34-160-0, 1:250). Then, samples were washed with 0.1% Triton X-100/PBS and incubated with Alexa Fluor®-conjugated secondary antibodies for 1 h at RT, and with the fluoroshield histology mounting medium with DAPI (Sigma–Aldrich, #F6057).

We took images from 5 microscopic fields (×20 objectives) per sample with the same exposure time and light intensity. For quantitative analyses, we set the same threshold limits for each marker. The mean of fluorescence intensities of γH2AX and phospho-E2F1 in each region of interest was quantified with ImageJ software. The fluorescence intensity from the background of each picture was subtracted from the corresponding values of the region of interest in the same image.

Images from fixed cultured cells were taken with a Thermo Scientific Invitrogen EVOS FL Auto 2 Imaging System (Fisher Scientific, #AMAFD2000), and images from fixed microvessel fractions were taken with an all-in-one fluorescence microscope (Keyence, #BZ-X810).

### 2.7. Cell Viability Assay

We used the CellTiter-Blue Cell Viability Assay (Promega, #G8080). The CellTiter-Blue reagent was added to the cell medium (1:5 ratio) with the cells. Cells were then incubated for 4 h at 37°C, and the fluorescence emission from the product resorufin was measured in a multiplate reader (560_Ex_/590_Em_).

### 2.8. Transendothelial Electrical Resistance (TEER) Measurement

CECs from 20-m/o mice were plated on the top inserts of Corning Transwell 24-well plates (50,000 cells/insert) with complete endothelial medium (250 µL in top insert and 700 µL in bottom compartment) and maintained until the TEER values maintained constant (3–4 days after plating). We used the EVOM2-Epithelial Voltohmmeter 2 (0.10K Ohms), following the manufacturer's instructions. TEER values were represented as Ohms/cm^2^.

### 2.9. Immunostaining of Brain Tissue

Four male and four female mice were transcardially perfused with cold PBS, and their brains were removed and further fixed by immersion in 4% paraformaldehyde overnight at 4°C. Brains were then immersed in a 30% sucrose/DPBS solution for 2 days at 4°C. We sliced mouse brains at 24 µm thickness with a freezing microtome. Then, sections were stored in an antifreezing solution at −20°C until needed.

For E2F1 staining, brain slices were incubated with 3% H_2_O_2_ for 30 min, blocked with 2% donkey serum/PBS for 30 min, and then incubated with anti-E2F1 (Santa Cruz Biotechnology, #sc-251, 1:100) overnight at 4°C. Then, samples were incubated with antimouse for 1 h and developed with the 3,3′-diaminobenzidine (DAB) substrate kit (Vector Laboratories, SK-4100). Slices were counterstained with hematoxylin for 3 min and dehydrated with an increased concentration of ethanol (70%–100%) and xylene. An investigator blinded to the experimental groups took the images from immunohistochemistry studies with an All-in-One Fluorescence Keyence Microscope. Four slides per brain were used, and we took images from 5 microscopic fields (×20 objectives) from each slide per brain. We used the color deconvolution command and the vector H DAB in ImageJ/Fiji software to separate color channels of 20x magnification images. The same threshold was applied to the brown channel to identify the E2F1-positive particles.

For γH2AX and CD31 staining, brain slices were incubated with a blocking buffer (5% BSA and 0.1% Triton X-100 in PBS) for 1 h at RT. Then, slices were incubated with anti-CD31 (Abcam, #ab28364, 1:250) and anti-γH2AX (Millipore, #05-636) dissolved in a blocking buffer overnight at 4°C. Slices were then washed with 0.1% Triton X-100 twice for 5 min at RT, and incubated with Alexa-conjugated secondary antibodies for 1 h at RT. Slices were washed twice and then mounted with fluoroshield mounting medium with DAPI. A blinded investigator to the experimental groups took the images with a Leica DMi8 microscope system. Four slides per brain were used, and we took images from 5 microscopic fields (×20 objectives) from each slide per brain. The color channels were split, and the red channel was used to identify the CD31-positive vessels using the same threshold in all images from all the groups. Then, the fluorescence intensity from the green channel of each image was quantified to analyze the levels of γH2AX in the brain vessels.

### 2.10. Behavior Tests

6–12 mice per group were tested for different behavior tests, as described next:

*Y*-maze: The mouse was placed in the intersection of a *Y*-shaped structure (39.5 × 8.5 × 13 cm) and allowed to move freely for 5 min. The movements of the mouse were digitally recorded, and an investigator blinded to the experimental groups analyzed the number of arm entries. The percentage of spontaneous alternation was calculated as ([number of alternations]/[total arm entries − 2]) × 100.

Open field: The mouse was placed in the middle of a square arena (40 cm per side) and allowed to explore for 10 min. Movements were digitally recorded. The Ethovision XT software automatically calculated velocity, distance moved, and the frequency and time spent in the center and borders of the arena.

Novel object recognition: A mouse in the arena was presented with two identical objects and allowed to explore for 10 min. 24 h later, one of these objects was replaced with a novel object, and the mouse was again allowed to explore for 10 min. We evaluated the differences in the exploration time with novel and familiar objects by calculating the recognition index as ([time with novel object]/[time with novel object + time with familiar object]).

Fear conditioning test: The mouse was placed in an arena (30 × 20 cm) and allowed to explore for 2 min. 1 h later, the animal was again placed in the arena. After 2 m, a “beep” sound (conditioning stimulus) was followed by a 1 mA electric foot shock (aversive stimulus) for 2 s. 24 h after the aversive stimulus, the animal was returned to the arena. After 1 min, the same “beep” sound occurred without an electric shock. Mouse movements were digitally recorded, and we analyzed the percentage of inactive time.

### 2.11. Statistics

We used GraphPad Prism software (v.10) to perform statistical analysis. We used Student's *t*-test to compare means from two independent groups and one-way ANOVA to compare more than two groups. Tukey's test was used for multiple comparisons. A value of *p*  < 0.05 was considered significantly different. Bar graphs represent mean ± SEM.

## 3. Results

### 3.1. E2F1 Was Downregulated in the Brains and the Brain Vasculature of Aged Mice Compared With Young Mice

**Figure 1:**
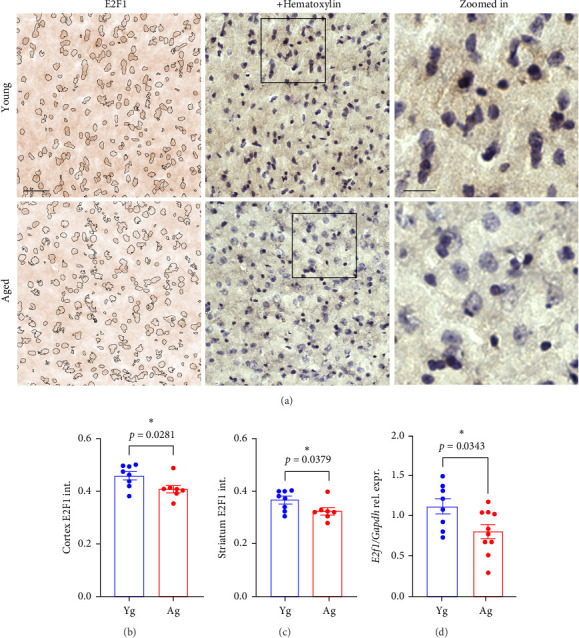
E2F1 was downregulated in the brains of aged mice compared with young mice. (A) Representative images of the cortex of 4- (young) and 20-m/o (aged) male mice processed for immunohistochemistry and stained with antibodies against E2F1 (brown) and counterstained with hematoxylin (purple). Hematoxylin-positive particles are identified in the left panel to visualize differences in E2F1 signal between young and aged groups. Scale bar, 100 µm. Zoomed-in scale bar, 50 µm. Quantification of E2F1 intensity from the cortices (B) and striatum (C) tissues from young and aged male and female mice. Data are mean ± SEM from 4 male and 4 female mice pooled together per age. Student's *t*-test. (D) Bar graph representing gene expression of *E2f1* relative to *Gapdh* in the brains of 4- (Yg) and 20-m/o (Ag) male and female mice. Data are mean ± SEM from 8 to 9 mice/age. Student's *t*-test.

First, we determined whether E2F1 was downregulated with aging in the mouse brains. We processed brain samples from young (4-m/o) and aged (20-m/o) mice for immunohistochemistry and found that the levels of E2F1 were significantly reduced in the cortex and striatum of aged mice compared with those in young mice (Figure 1A–C). We also determined the relative gene expression of *E2f1* and found that the brains of aged mice had a significantly reduced relative expression of *E2f1* compared with brains from young mice (Figure 1D). This indicates that aging downregulates the expression and protein levels of E2F1 in the brains of mice.

**Figure 2:**
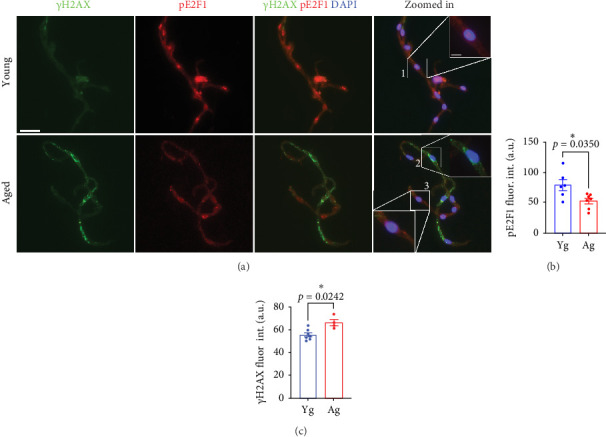
Phospho-E2F1 and γH2AX were reduced and increased, respectively, in microvessel fractions from aged mice compared with young mice. (A) Representative images of microvessel fractions isolated from 4- (young) and 20-m/o (aged) male mice and immunostained against γH2AX (green) and phosphorylated form E2F1 (pE2F1, red) and the nuclear DAPI dye (blue). Scale bar, 20 µm. Zoomed-in scale bar, 5 µm. (B) Quantification of fluorescence intensity of phospho-E2F1 from (A). (C) Quantification of fluorescence intensity of γH2AX from (A). Note that the cells with higher fluorescence intensity of phospho-E2F1 have lower fluorescence signals from γH2AX. Data are mean ± SEM from 4 to 7 mice/age. Student's *t*-test.

E2F1 regulates the expression of multiple genes involved in DNA repair, and it maintains genome integrity [15–18]. Our major goal in this study was to determine whether E2F1 is associated with DNA damage in the brain endothelium. We hypothesized that aged mice may show a reduction in E2F1 and consequently have increased DNA damage in their brain endothelium compared with young mice. Thus, we isolated microvessel fractions from the brains of young (4-m/o) and aged (20-m/o) mice. Figure S1 shows the characterization of these microvessels, which are positive for CD31 and show expression of the gene *Chd5* that codes for cadherin-5, a marker of endothelial cells. The microvessel fractions show minimal expression of *Aqp4*, *Cspg4*, *Mog*, and *Nefl*, which are genes coding for markers of astrocytes, oligodendrocyte precursor cells, mature oligodendrocytes, and neurons, respectively. We used antibodies against phosphorylated E2F1 (Ser375), which is associated with its transcriptional activity [42–44]. Microvessels were stained with antibodies against phospho-E2F1(Ser375) and the DNA damage marker γH2AX. γH2AX is a phosphorylated form of the histone H2AX at Ser139. The phosphorylation of H2AX is the first event after a DNA insult that triggers the recruitment of different DNA repair factors and activates signaling pathways to repair DNA damage [45]. We found that phospho-E2F1 was significantly reduced in brain microvessels isolated from aged mice compared with microvessels from the brains of young mice (Figure 2B) and that γH2AX was significantly increased in the brain microvessel fraction of aged mice, compared with young mice (Figure 2C). Indeed, those endothelial cells with higher phospho-E2F1 fluorescence signals had reduced γH2AX fluorescence intensity, indicating that increased activity of E2F1 protects endothelial cells from DNA damage. Interestingly, we observed that the immunostaining with anti-γH2AX occurred not only in the nuclei of cells but also perinuclearly. *γ*H2AX also occurs in the DNA of mitochondria under oxidative stress [46]. Thus, the perinuclear staining may be due to mitochondrial DNA damage, which may accumulate in the brain endothelium with aging.

**Figure 3:**
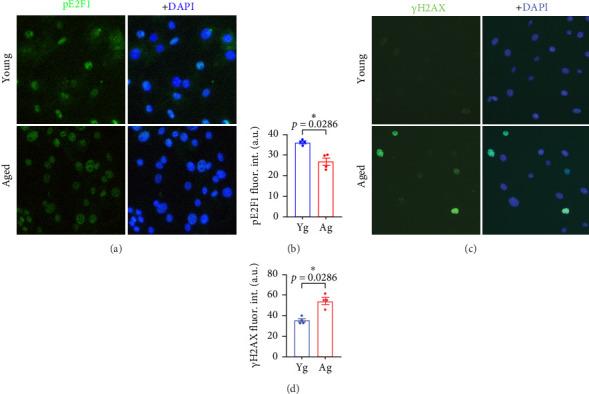
Cultured aged mouse-derived CECs showed reduced phospho-E2F1 and enhanced DNA damage compared with young mouse-derived CECs. (A) Representative images of cultured CECs isolated from 4- (young) and 20-m/o (aged) male mice stained with antibodies against phospho-E2F1 (pE2F1, green) and the nuclear DAPI dye (blue). Scale bar, 50 µm. (B) Quantification of fluorescence intensity of phospho-E2F1 from (A). (C) Representative images of CECs stained with antibodies against γH2AX (green) and the nuclear DAPI dye (blue). Scale bar, 50 µm. (D) Quantification of fluorescence intensity of γH2AX from (C). Data are mean ± SEM from independent cultures of CECs from four mice/age. Student's *t*-test.

Next, we used a primary culture of endothelial cells isolated from the brains of adult male mice. Previously, we demonstrated that this *in vitro* model shows minimal contamination with other brain cell types and conserves important characteristics observed in the brain vasculature of mice [38]. Indeed, our culture of CECs maintains features associated with the animals from which the cells were derived [38, 39]. We isolated and cultured primary CECs from 4- and 20-m/o mice. We observed that cultured primary CECs derived from aged mice showed reduced phospho-E2F1 compared with CECs from young mice (Figure 3A,B). This recapitulates the reduced phosphorylation of E2F1 that we observed in the microvessel fractions from aged mice, compared with young mice (Figure 2). In addition, CECs derived from aged mice, compared with young mice-derived CECs, showed enhanced DNA damage, as indicated by increased fluorescence intensity of the DNA damage marker γH2AX (Figure 3C,D). Thus, this data indicates that the reduced phosphorylation of E2F1 that occurs with aging in cultured mouse-derived CECs is accompanied by enhanced DNA damage, which supports our finding in brain microvessels.

### 3.2. *E2f1* Overexpression Reduced DNA Damage in Cultured CECs Derived From Aged Mice

**Figure 4:**
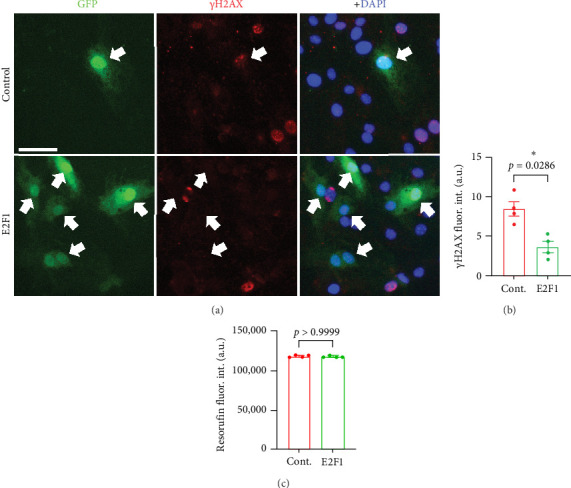
*E2f1* overexpression reduced DNA damage in cultured aged-mouse-derived CECs. (A) Cultured CECs from 20-m/o male mice were transfected either with an empty plasmid and pCAG-GFP (control), or with pCMV-HA-E2F1 and pCAG-GFP (E2F1). Then, cells were fixed and stained with antibodies against γH2AX (red) and the nuclear DAPI dye (blue). Scale bar, 50 µm. (B) Quantification of fluorescence intensity of γH2AX from (A). (C) Quantification of the fluorescence intensity CellTiter-Blue (resorufin) in cultured CECs transfected either with an empty plasmid and pCAG-GFP (cont.), or either with pCMV-HA-E2F1 and pCAG-GFP (E2F1). Measurement was performed 72 h after transfection. Data are mean ± SEM from independent cultures of CECs from four mice. Student's *t*-test.

E2F1 participates in DNA repair pathways to maintain genome integrity [15–18]. Next, we hypothesized that upregulating E2F1 can reduce DNA damage in cultured primary CECs isolated from aged mice. We isolated and cultured CECs from 20-m/o mice. Cells were transfected either with an empty plasmid (pCMV-HA) and a plasmid coding for GFP (pCAG-GFP), as control cells, or a plasmid coding for E2F1 (pCMV-HA-E2F1) and the GFP plasmid, as E2F1 cells. We used the GFP plasmid to visualize those cells that were positively transfected. Using flow cytometry, we determined that the transfection efficiency of these cells was around 35% (Figure S2A,B). We found that the transfection procedure does not significantly affect the proliferation of cultured CEC, compared with nontransfected cells (Figure S2C). Then, to determine if *E2f1* overexpression reduced DNA damage, control and E2F1 cells were fixed and stained with antibodies against γH2AX. We observed that the fluorescence intensity of γH2AX was reduced in CECs overexpressing E2F1, compared with cells transfected with the control plasmid (Figure 4A,B). The overexpression of *E2f1* did not reduce the proliferation of CECs (Figure 4C). Thus, our data indicate that *E2f1* overexpression reduces DNA damage in cultured CECs isolated from aged mice.

### 3.3. *E2f1* Overexpression in the Brain Vasculature of Aged Mice Improved Spatial Memory and Contextual Learning, Reduced DNA Damage, and Increased the Expression of BBB-Associated Genes

**Figure 5:**
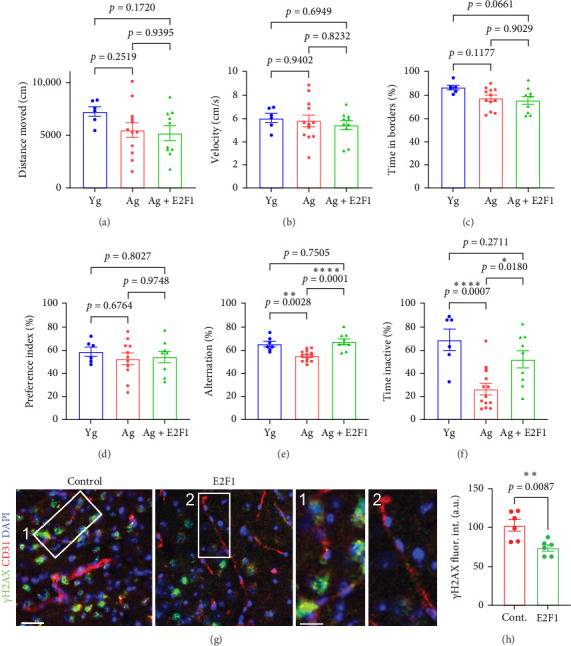
*E2f1* overexpression in the brain vasculature improved spatial memory and contextual learning and reduced DNA damage in aged mice. 20-m/o male mice were injected (i.v.) with AAV (BR1)-CAG-mE2F1-T2A-GFP (E2F1) or AAV (BR1)-CAG-T2A-GFP, as control. 4-m/o mice were injected with AAV (BR1)-CAG-T2A-GFP as control young mice. Two months after injection, mice were tested for open field (A–C), *Y*-maze (D), novel object recognition (E), and fear conditioning (F). (A) Distance moved, (B) velocity, (C) percentage of time spent in borders of the arena, (D) preference index, (E) percentage of alternation, and (F) percentage of time inactive. Data are mean ± SEM from 6 to 12 mice/group. One-way ANOVA test, Tukey's multiple comparisons test. (G) Representative images of the cortex of 20-m/o mice previously injected with the control vector, or with the vector coding for E2F1. Brain slices were stained with antibodies against γH2AX (green) and CD31 (red). Scale bar, 50 µm. Zoomed-in scale bar, 25 µm. Note that the cells in CD31-positive vessels in the zoomed in Image 2 (E2F1 mouse) show reduced γH2AX fluorescence than in cells in the zoomed in Image 1 (control). (H) Quantification of fluorescence intensity of γH2AX from (G). Data are mean ± SEM from 5 to 6 mice per type of vector. Student's *t*-test.

Given that phospho-E2F1 was reduced in the microvessel fractions of aged mice and that *E2f1* overexpression reduced DNA damage in cultured CECs derived from aged mice, we sought to determine if upregulating *E2f1* in the brain vasculature of aged mice could also reduce DNA damage in the brain endothelium and have beneficial effects on cognitive function. For this study, we used AAV capsid variant BR1, which shows high tropism for the cerebral endothelium and durable expression (>660 days) [37]. We designed the vector AAV (BR1)-CAG-mE2F1-T2A-GFP to overexpress *E2f1* preferentially in the brain endothelium of mice. We also designed the vector AAV (BR1)-CAG-T2A-GFP as a control construct. 4-m/o and 20-m/o were injected with the control vector, and another group of 20-m/o mice was injected with AAV (BR1)-CAG-mE2F1-T2A-GFP. We confirmed that the vectors extensively targeted the mouse brain vasculature (Figure S3). Two months after injection, mice were tested for open field, *Y*-maze, novel object recognition, and fear conditioning. In the open field, we determined the distance moved (Figure 5A), velocity (Figure 5B), and the percentage of time spent in borders (Figure 5C). We found no significant differences in these parameters between *E2f1*-expressing aged mice and aged control mice, indicating that *E2f1* overexpression in the brain vasculature did not affect either overall motor activity nor did it produce hyperactivity or anxiety-like phenotype. We did not observe significant changes in the preference index by novel object recognition (Figure 5D), indicating that the recognition memory was not regulated by *E2f1* overexpression in the brain vasculature. However, targeting the brain vasculature of aged mice to overexpress *E2f1* did increase the percentage of alternation by *Y*-maze (Figure 5E) and the percentage of time inactive by fear conditioning (Figure 5F), indicating that *E2f1* overexpression in the brain endothelium improved both spatial memory and contextual learning in aged mice. To confirm that *E2f1* overexpression reduced DNA damage in the brain endothelium, brain slices from the experimental mice were stained with antibodies against CD31 to visualize the brain vasculature and antibodies against the DNA damage marker γH2AX. We observed that the vessels in mice injected with the E2F1 vector showed reduced γH2AX fluorescence intensity compared with the vessels in control mice (Figure 5G,H). This indicates that *E2f1* overexpression reduces DNA damage in the brain vasculature of aged mice.

**Figure 6:**
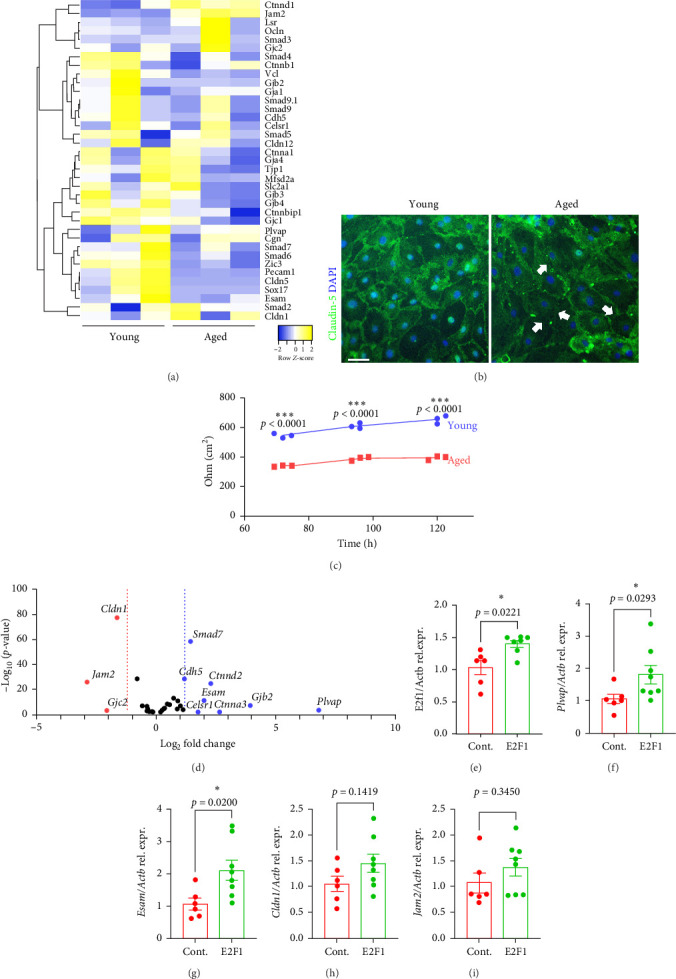
*E2f1* overexpression in the brain endothelium increased the expression of genes associated with the BBB in mice. (A) From an mRNA sequencing analysis from cultured CECs isolated from young and aged mice, the heatmap represents the differential expression of genes involved in BBB and vascular permeability. (B) Representative images of cultured CECs isolated from 4-m/o (young) and 20-m/o (aged) mice and immunostained with claudin-5 (green) and the nuclear DAPI dye (blue). Scale bar, 100 µm. Note that the claudin-5 signal is reduced and discontinuous in the monolayer culture of CECs isolated from aged mice, compared with claudin-5 in cultured CECs from young mice. (C) Transendothelial electrical resistance values per area (cm^2^) of cultured CECs isolated from young and aged mice 72, 96, and 120 h after plating cells on the inserts. Data are mean ± SEM from independent cultures of CECs isolated from 3 mice per age. Student's *t*-test per each time point between young and aged CECs. (D) Volcano plot showing fold changes for genes involved in BBB integrity and permeability differentially expressed between control CECs versus *E2f1*-overexpressing CECs in culture. (E–I) Gene expression of genes of interest relative to β-actin in the brains of mice previously injected with the control vector or the vector coding for E2F1. These genes are *E2f1* (B), *Plvap* (C), *Esam* (D), *Cldn1* (E), *Jam2* (F). Data are mean ± SEM from 6 to 8 mice per type of vector. Student's *t*-test.

Next, we wondered how *E2f1* overexpression, particularly in the brain vasculature, can improve memory in aged mice. Genome instability in the brain endothelium contributes to vascular dysfunction and negatively affects the integrity of the brain vasculature, which increases nonselective permeability and enhances leakage from the BBB [7, 8]. We explored this possibility by analyzing the expression of genes involved in vascular integrity. Given that our model of cultured CECs recapitulates many features observed in the brain vasculature of mice, we used mRNA sequencing data from cultured CECs isolated from young and aged mice that were previously published by our lab [38]. The representation of the expression of genes associated with BBB integrity and permeability [47] in a heatmap revealed that there were notable differences between cultured CECs from young and aged mice (Figure 6A). To elucidate if these differences in gene expression corresponded to differences in vascular integrity, CECs from young and aged mice were isolated, cultured, fixed, and stained with antibodies against the tight junction protein claudin-5. The immunostaining showed that the claudin-5 signal was more uniform and continuous in CECs from young mice than in CECs from aged mice (Figure 6B). We also determined that there existed significant differences in the TEER values between cultured CECs from young and aged mice (Figure 6C). Overall, these data indicate that cultured CECs from aged mice showed deficits in vascular integrity compared with CECs derived from young mice.

Next, to explore the possibility of a role of E2F1 on vascular permeability, we identified the genes coding for junctional proteins in the BBB [47] that were regulated differentially between aged mice-derived CECs transfected with a control plasmid and the CECs transfected with a plasmid coding for *E2f1*. A volcano plot represents the BBB-associated genes, such as *Plvap*, *Gjb2*, *Ctnna3*, and *Esam*, that were significantly upregulated in *E2f1*-overexpressing CECs, compared with control cells (Figure 6D). However, genes, such as *Jam2*, *Cldn1*, and *Gjc2* were downregulated. This data indicates that *E2f1* overexpression mostly upregulates the expression of genes that contribute to the selective permeability of the BBB.

Then, we aimed to confirm if the effect of overexpressing *E2f1* in cultured CECs also occurs when *E2f1* was overexpressed in the brain endothelium of aged mice. Thus, we analyzed the expression of genes coding for junctional proteins in the BBB in the brain lysates of control mice and *E2f1*-overexpressing mice. We confirmed that *E2f1* was significantly upregulated in the brains injected with the E2F1 vector, compared with control mice (Figure 6E). We found that the brains from mice injected with the E2F1 vector showed significantly increased expression of *Plvap* and *Esam* (Figure 6F,G). Interestingly, we did not find significant differences in the expression of *Cldn1* and *Jam2* (Figure 6H,I), which were downregulated in *E2f1*-overexpressing CECs. Our data suggest that *E2f1* overexpression improves the integrity of the brain endothelium in aged mice.

### 3.4. *E2f1* Overexpression Significantly Upregulated the Expression of *Acod1*

We observed that brain tissues from mice injected with the AAV (BR1)-CAG-T2A-GFP vector contained some neuron-shaped cells that expressed GFP (Figure S3B). Another study reported that the AAV (BR1) vector can transduce neurons in mice [48]. *E2f1* overexpression can promote apoptosis in neurons [32, 49]. Thus, the use of tools to overexpress *E2f1* in the brain has to be considered carefully. Instead, identifying downstream gene targets of E2F1 would avoid the risk of overexpressing *E2f1* in neurons and promote potential neurodegeneration.

**Figure 7:**
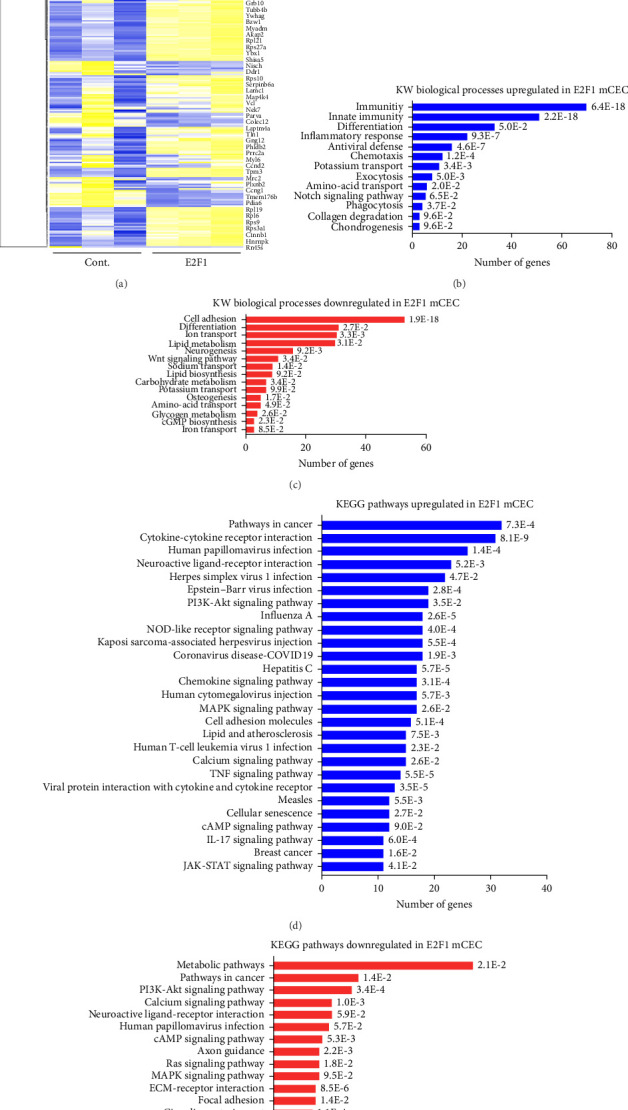
*Acod1* was significantly upregulated by *E2f1* overexpression in cultured aged mouse-derived CECs. (A) Cultured CECs isolated from 20 m/o male mice were transfected with pCMV-HA-E2F1 (E2F1) or pCAG (cont.). Then, RNA from cells was isolated and processed for RNA sequencing analysis. The heatmap shows the 500 most expressed genes in control and *E2f1*-overexpressing CECs. Representation of the biological processes upregulated (B) and downregulated (C) in *E2f1*-overexpressing CECs compared with control CECs. Each biological process shows a *p* value. Representation of the most enriched KEGG pathways containing the most significant upregulated (D) and downregulated (E) genes in *E2f1*-overexpressing CECs, compared with control CECs. Each KEGG pathway shows a *p* value. (F) Volcano plot showing fold changes for genes differentially expressed between control CECs versus *E2f1*-overexpressing CECs in culture. (G) Gene expression of *E2f1* relative to β-actin in cultured CECs transfected with a control plasmid (cont.) or with a plasmid coding for *E2f1* (E2F1). (H) Gene expression of *Acod1* relative to β-actin in cultured CECs transfected with a control plasmid (cont.) or with a plasmid coding for *E2f1* (E2F1). Data are mean ± SEM from independent cultures of CECs from three aged mice. Student's *t*-test.

To identify E2F1 downstream targets that can prevent DNA damage in brain endothelial cells, we cultured CECs derived from 20-m/o mice and transfected them with a control plasmid and a plasmid coding for GFP (control cells) or a plasmid coding for E2F1 and the GFP plasmid (E2F1 cells). Then, we analyzed their transcriptome by RNA sequencing. Analyses of the 500 most expressed genes revealed important changes in the expression of most of these genes (Figure 7A). *E2f1* overexpression in cultured CECs upregulated biological processes associated with immunity, differentiation, and inflammatory response (Figure 7B). *E2f1* overexpression downregulated biological processes associated with cell adhesion, differentiation, and ion transport (Figure 7C). Pathway enrichment analysis informed that *E2f1* overexpression upregulated the expression of genes involved in pathways in cancer, cytokines interaction, virus infection, and PI3K-Akt signaling, among others. However, *E2f1* overexpression downregulated the expression of genes involved in metabolic pathways, pathways in cancer and infection, and signaling pathways of PI3K-Akt, calcium, and cAMP, among others (Figure 7E). We identified that *E2f1* overexpression significantly upregulated the expression of immune response genes, such as *Rsad2*, *Cxcl9*, *Il1rl1*, *Oasl1*, *CCl5*, and *Acod1;* the latter was the gene with the highest fold change (Figure 7F). We confirmed that E2F1 cells had significantly increased expression of *E2f1* and *Acod1* in cultured primary CECs derived from aged mice (Figure 7G,H).

### 3.5. 4-OI Prevented DNA Damage, Promoted Cell Proliferation, and Restored Endothelial Barrier Function After Oxidative Stress in Cultured Mouse-Derived CECs

**Figure 8:**
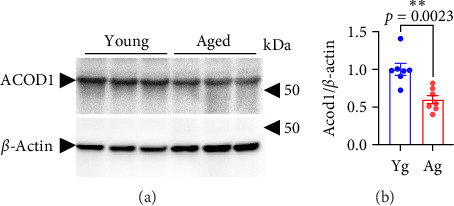
ACOD1 levels were reduced in the brains of aged mice compared with young mice. (A) Representative image of a Western blot for ACOD1 and the housekeeping protein β-actin from the brain lysates of 4-m/o (young) and 20-m/o (aged) mice and (B) bar graph representing the band intensity of ACOD1 relative to the intensity of the correspondent β-actin. Data are mean ± SEM from seven mice. Student's *t*-test.

*Acod1* codes for aconitate decarboxylase-1 (ACOD1), an enzyme that synthesizes itaconate in mitochondria. Itaconate is produced by decarboxylation of cis-itaconate in the tricarboxylic acid cycle (aka the Krebs cycle) [50]. Itaconate has anti-inflammatory and antioxidant effects through multiple mechanisms in different organisms [51–53]. We found that E2F1 is downregulated in the brains of aged mice compared with young mice (Figure 1) and that *Acod1* was upregulated in *E2f1*-overexpressing CECs (Figure 7). Thus, we hypothesize that ACOD1 levels are reduced in the brains of aged mice compared with young mice. We found that ACOD1 was significantly reduced in the mouse brains with aging (Figure 8).

Itaconate has anti-inflammatory and antioxidative properties. Thus, we hypothesized that treating cultured CECs derived from aged mice with itaconate can mitigate or prevent DNA damage. For this, we used a derivative form of itaconate, 4-OI, that is cell-permeable and has been used in *in vitro* and *in vivo* models to inhibit oxidative stress [54–56].

**Figure 9:**
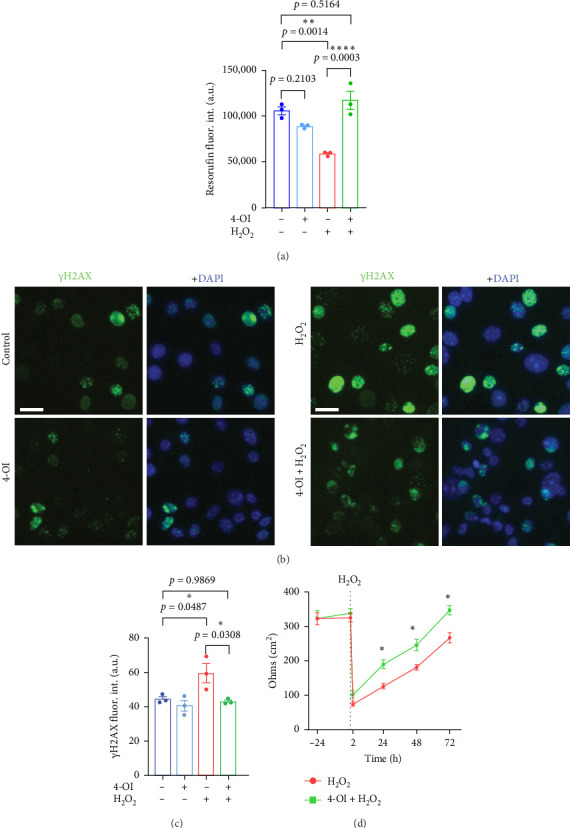
A derivative itaconate protected cultured CECs from oxidative stress. (A) Quantification of the fluorescence intensity CellTiter-Blue (resorufin). Cultured CECs were treated with 10 µM 4-OI (1 h) or a vehicle and then treated with 500 µM H_2_O_2_ (2 h) or a vehicle. The medium was replaced with fresh complete endothelial medium, and cells were maintained for 24 h. The CellTiter-Blue reagent was added, and fluorescence was measured. Data are mean ± SEM from cultured CECs isolated from three 20-m/o mice. One-way ANOVA test, Tukey's multiple comparisons test. (B) Representative images of cultured CECs derived from aged mice stained with anti-γH2AX and DAPI. Cultured aged mouse-derived CECs were treated with 10 µM 4-OI (1 h) or a vehicle and then treated with 500 µM H_2_O_2_ or a vehicle for 2 h. The medium was replaced, and cells were maintained for 24 h. Then, cells were fixed and stained with antibodies against γH2AX (green) and with the nuclear DAPI dye (blue). Scale bar, 25 µm. (C) Quantification of the fluorescence intensity of γH2AX from (B). Data are mean ± SEM from independent cultures of CECs from three aged mice. One-way ANOVA test, Tukey's multiple comparisons test. (D) Transendothelial electrical resistance values per area (cm^2^) of cultured CECs isolated from aged mice plated in the inserts of transwell plates. Cells were pretreated with 10 µM 4-OI or a vehicle for 1 h, and then treated with 500 µM H_2_O_2_ or a vehicle for 2 h. The medium was replaced with complete endothelial medium, and cells were maintained until 72 h. Data are mean ± SEM from independent cultures of CECs from four aged mice. Student's *t*-test per each time point between H_2_O_2_ and 4-OI + H_2_O_2_.

Cultured CECs isolated from 20-m/o were pretreated with different concentrations of 4-OI (10, 25, and 50 µM) or a vehicle for 1 h. Then, cells were treated with H_2_O_2_ (500 µM) for 2 h to induce oxidative stress or H_2_O as the vehicle. The medium was replaced, and cells were maintained for 24 h. Then, cells were incubated with CellTiter-Blue reagent for 4 h to determine cell proliferation. We observed that treating CECs with 500 µM H_2_O_2_ significantly reduced cell proliferation. However, the pretreatment of CECs with 10 µM 4-OI prevented the harmful effect of H_2_O_2_ on cultured CECs (Figure 9A and Figure S4). Furthermore, to determine if 4-OI indeed protects DNA integrity, cultured CECs from aged mice were pretreated with 10 µM 4-OI or a vehicle for 1 h, and then treated with 500 µM H_2_O_2_or H_2_O as a vehicle. Cells were fixed and stained with antibodies against γH2AX. We measured the fluorescence intensity of γH2AX and found that H_2_O_2_ significantly increased the levels of γH2AX, and that treating CECs with 4-OI prevented DNA damage induced by H_2_O_2_ in cultured CECs (Figure 9).

*E2f1* overexpression increased the expression of genes involved in vascular permeability and endothelial cell interaction, suggesting that E2F1 can improve the integrity of the BBB. We wondered if itaconate may have beneficial effects on vascular permeability in aged CECs. For this, cultured CECs isolated from aged mice were plated in transwell inserts and pretreated with 4-OI (10 µM) or a vehicle for 1 h. Then, the medium was replaced with fresh complete medium, and cells were incubated for 72 h. TEER was measured 24 h before H_2_O_2_ treatment, right before H_2_O_2_, 2 h after H_2_O_2_, and 24, 48, and 72 h after replacing the medium. We observed that CECs pretreated with 4-OI recovered the original TEER values faster than cells not treated with 4-OI (Figure 9F). Overall, our data indicate that 4-OI has beneficial effects on cultured CECs derived from aged mice by protecting DNA, promoting cell proliferation, and restoring endothelial barrier function from oxidative stress induced by H_2_O_2_.

## 4. Discussion

Our study demonstrates the role of E2F1 in reducing DNA damage in brain endothelial cells, and it is the first study that demonstrates that targeting the brain vasculature to upregulate *E2f1* improves cognition in aged mice, likely due to increased expression of genes associated with junctional functions of the BBB. This is also the first study that indicates that *Acod1* is upregulated in *E2f1*-overexpressing endothelial cells and that a derivative itaconate prevents DNA damage, promotes cell proliferation, and restores endothelial barrier function from oxidative stress in cultured CECs.

We found that *E2f1* overexpression reduced DNA damage in aged mouse-derived CECs. E2F1 can maintain the integrity of the genome by binding to DNA damage sites and recruiting DNA repair factors [15–18]. We also found that preferentially targeting the brain vasculature with AAV serotype BR1 to overexpress *E2f1* improved spatial memory and contextual fear learning. However, the literature shows both beneficial and deleterious effects of the manipulation of E2F1 in endothelial cells. On one hand, several studies claim that *E2f1* overexpression in different cell lines of cultured endothelial cells promotes cell viability, migration, angiogenesis [57–60], and tube formation [61]. In mice, *E2f1* overexpression at sites of carotid balloon injury promotes functional endothelial recovery in a rat model of balloon angioplasty [62]. On the other hand, *E2f1* downregulation prevents endothelial dysfunction induced by high glucose in cultured HUVEC [63], and E2F1 depletion in bone marrow progenitor cells results in increased mitochondrial respiration and enhanced endothelial differentiation [64]. *E2f1* depletion in mice results in endothelial cell proliferation and reduced apoptotic endothelial cells, compared to WT mice [26]. The transplantation of *E2f1*-depleted bone marrow progenitor cells in irradiated WT mice increased the differentiation of bone marrow cells into endothelial cells [64]. Furthermore, *E2f1*-null mice show enhanced vessel density and increased endothelial cell proliferation in the border zone of a wound, which helps skin heal faster compared with WT mice [23]. This dual role of E2F1 in endothelial cells may likely depend on the type of endothelial tissue, stimulus, and environment. Thus far, no studies have tested the role of E2F1 by targeting the brain vasculature. In addition, no studies have been performed using aged mice. Thus, the significance of our study relies on the specificity of targeting the brain vasculature using aged mice.

We found that *E2f1* overexpression in cultured CECs significantly upregulated the expression of the gene *Acod1. Acod1* codes for the enzyme ACOD1, which synthesizes itaconate or itaconic acid from cis-aconitate in the tricarboxylic acid cycle in mitochondria. Itaconate is a potent inhibitor of bacterial growth. From a molecular perspective, the beneficial effects of itaconate are mediated, at least, by activation of the transcription factors Nrf2 and Atf3 [53], and by the inhibition of succinate dehydrogenase [51]. During the early 20^th^ century, the role of itaconate in mammalian metabolism was considered very unlikely. However, in 2011, the implication of itaconate as an immunoregulatory metabolite gained more prominence. In 2013, Michelucci et al. [65] discovered that itaconate is produced by the enzyme coded by the gene *Acod1*, whose expression is aberrantly upregulated in mouse macrophages during LPS-induced inflammation, reaching up to 200-fold change in expression, making it the most highly inducible gene. *Acod1* is mainly expressed in myeloid cells, including activated macrophages, but there is evidence that it can also be upregulated in non-immune cells, such as mouse lung endothelial cells [66]. Our study supports that *Acod1* can be upregulated in non-immune cells, as it is highly expressed in *E2f1*-overexpressing mouse CECs. Interestingly, Pan et al. [67] found that ACOD1 knockdown reduced the protein levels of E2F1 in glioma cells. This evidence, in conjunction with our data, suggests that E2F1 and ACOD1 can be regulated by each other in a positive feedback loop.

Itaconate is a compound of the Krebs cycle with anti-inflammatory properties. However, circulatory itaconate is rapidly removed from the body of rats within 24 h after administration, and its effects on mitochondrial respiration last for only 3–4 h [68]. This makes the use of itaconate a nonoptimal therapeutic tool [69]. Thus, different derivatives of itaconate have been formulated to enhance cell permeability and molecule delivery, such as 4-OI and dimethyl-itaconate. For example, dimethyl-itaconate decreases neurological deficit in a mouse model of cerebral ischemia-reperfusion injury [70]. However, these derivatives also have some limitations: dimethyl-itaconate is not metabolized into itaconate inside cultured cells [71], and 4-OI and dimethyl-itaconate are more reactive than itaconate, dimethyl-itaconate being the most reactive of both derivatives [69]. Despite the limitations of the itaconate derivatives, it has been demonstrated that 4-OI has beneficial effects on *in vitro* and *in vivo* models. 4-OI reduces oxidative stress, inflammatory response, and lung injury in a mouse model of mechanical ventilation [56]. Similarly, in cultured primary mouse lung vascular endothelial cells, 4-OI pretreatment inhibits oxidative stress and inflammatory response induced by cyclic stretch [56]. The use of a hydrogel with 4-OI combined with polyethylene glycol, which enhances molecule delivery, improves blood vessel regeneration and perfusion, accelerates wound healing, reduces the levels of proinflammatory molecules at the wound sites, and tissue regeneration in a diabetic wound mouse model [54]. The 4-OI hydrogel also inhibits oxidative stress, prevents mitochondrial integrity and membrane potential, and promotes neovascularization in cultured human umbilical vein endothelial cells [54]. 4-OI also inhibits oxidative stress induced by high glucose in cultured human umbilical vein endothelial cells [55]. However, dimethyl-itaconate inhibits cell growth, cell tube formation, and cell migration in cultured bovine aortic endothelial cells and cultured human umbilical endothelial cells [72]. From this evidence, we used 4-OI in our *in vitro* model of CECs and found that pretreating cultured CECs derived from aged mice with 4-OI prevented DNA damage and promoted cell survival in H_2_O_2_-induced oxidative stress. Given that ROS production and oxidative stress are increased in aged brains and the brains of patients with neurodegenerative disorders [10], the use of itaconate derivatives or targeting ACOD1 may be a potential therapy to prevent or mitigate the pathophysiology of these disorders.

We found that overexpressing *E2f1* in the brain vasculature of aged mice increased the expression of the BBB-associated genes *Plvap* and *Esam*, and that treating cultured mouse-derived CECs with 4-OI restored TEER values after oxidative stress induced by hydrogen peroxide. *Plvap* codes for the protein plasmalemma vesicle-associated protein that is involved in the regulation of basal vascular permeability, and *Esam* codes for the protein endothelial cell adhesion molecule that mediates homophilic interactions between endothelial cells. This suggests that the improvement of cognition in mice by overexpressing *E2f1* in the brain vasculature may be caused by a restoration of the BBB function. The BBB regulates the flow of molecules, ions, and cells between the bloodstream and the brain parenchyma. However, with aging, the integrity of the BBB becomes dysfunctional, and the selective permeability of the BBB is disrupted, allowing the transport of neurotoxic compounds and peripheral immune cells into the brain tissue [73]. Many brain disorders, such as AD, Parkinson's disease, and amyotrophic lateral sclerosis, are characterized by dysfunctional BBB [74]. Thus, the identification of potential targets that protect or ameliorate the BBB integrity may hinder the progression of the pathology of these disorders. Thus far, the research on the role of E2F1 in the BBB function is very limited. Guo et al. [75] found that upregulating the E2F1-MDM2 axis reduces TEER values and downregulates the levels of the tight junction proteins occludin, VE-cadherin, and claudin-5 in brain endothelial cells isolated from neonatal rats. However, no previous studies have used *in vivo* models to determine the effects of *E2f1* overexpression in the brain vasculature. Given that E2F1 dysregulation has been associated with the progression of glioblastomas [76, 77], E2F1 as a target to improve the BBB and cognitive functions has to be considered very cautiously. Interestingly, we found that the expression of*Acod1* was upregulated in *E2f1*-overexpressing CECs and in the brains of aged mice compared with young mice. The beneficial effects of itaconate, the product of ACOD1, as an anti-inflammatory mediator have been recently recognized [78, 79]. Interestingly, *Acod1* deletion in a mouse model of stroke aggravates BBB disruption [80], and the treatment with dimethyl itaconate alleviates BBB disruption in mouse models of stroke mice [80] and autoimmune encephalomyelitis [81]. These studies support our finding that a derivative itaconate improves endothelial barrier function. Given that these investigations used young animals, future studies should address whether these effects also occur in aged mice.

## 5. Conclusions

Targeting preferentially the brain endothelium to overexpress the transcription factor E2F1 reduced DNA damage in the brain endothelium of aged mice, increased the expression of BBB-associated genes *Plvap* and *Esam*, and improved spatial memory and contextual learning. The gene that codes for the enzyme ACOD1, *Acod1*, was one of the most significantly upregulated genes in *E2f1*-overexpressing CECs. Using the cell-permeable form of itaconate, 4-OI, prevented DNA damage, promoted cellular proliferation, and restored endothelial barrier function in cultured CECs from oxidative stress.

## Figures and Tables

**Table 1:** Sequences of primers.

Gene name	Species	Fw/Rv	Primer sequence
*Actb*	Mouse	Fw	5′-CATTGCTGACAGGATGCAGAAGG-3′
		Rv	5′-TGCTGGAAGGTGGACAGTGAGG-3′

*E2f1*	Mouse	Fw	5′-GGATCTGGAGACTGACCATCAG-3′
		Rv	5′-GGTTTCATAGCGTGACTTCTCCC-3′

*Acod1*	Mouse	Fw	5′-GGTATCATTCGGAGGAGCAAGAG-3′
		Rv	5′-ACAGTGCTGGAGGTGTTGGAAC-3′

*Gapdh*	Mouse	Fw	5′-CAAGGTCATCCATGACAACTTTG-3′
		Rv	5′-GTCCACCACCCTGTTGCTGTAG-3′

*Plvap*	Mouse	Fw	5′-GTTGACTACGCGACGTGAGATG-3′
		Rv	5′-AGCTGTTCCTGGCACTGCTTCT-3′

*Esam*	Mouse	Fw	5′-GCAAGGCTCAAAACAGAGTGGG-3′
		Rv	5′-CAAAAGTGCCCACAACTGCTCC-3′

*Cldn1*	Mouse	Fw	5′-GGACTGTGGATGTCCTGCGTTT-3′
		Rv	5′-GCCAATTACCATCAAGGCTCGG-3′

*Jam2*	Mouse	Fw	5′-CAGACTGGAGTGGAAGAAGGTG-3′
		Rv	5′-GCTGACTTCACAGCGATACTCTC-3′

## Data Availability

The data obtained from this study are openly available in Harvard Dataverse at https://doi.org/10.7910/DVN/VMZCY1.

## Nomenclature

BBB: Blood–brain barrier
CECs: Cerebral endothelial cells
4-OI: 4-Octyl itaconate
ACOD1: Aconitate decarboxylase-1
m/o: Months old.

## Supporting Information

Supporting Information Description of the methods and figure legends of the supporting information figures. Supporting information Figure for submission-Final. Four supporting information figures include additional information to the main results in the manuscript.
