# Supplementary material for: E2f1 Overexpression Reduces Aging-Associated DNA Damage in Cultured Cerebral Endothelial Cells and Improves Cognitive Performance in Aged Mice
Source: Oxid Med Cell Longev. 2025 Jul 28;2025:3242282. doi: 10.1155/omcl/3242282 (PMC12321429; doi:10.1155/omcl/3242282)
Supplement: Supporting Information — Description of the methods and figure legends of the supporting information figures. Supporting information Figure for submission-Final. Four supporting information figures include additional information to the main results in the manuscript. [file 3242282.f1.docx]

**Supplementary material**

**Title: *E2f1* overexpression reduces aging-associated DNA damage in cultured cerebral endothelial cells and improves cognitive performance in aged mice.**

**Supplementary Methods**

Immunostaining of Cultured CEC and microvessel fractions

Cultured cells were fixed with a solution of 4% PFA/PBS for 10 m at RT, and permeabilized with 0.1% Triton-X100 for 10 m at RT. A solution of 5% bovine serum albumin in PBS was used as a blocking solution. Cells were blocked overnight at 4ºC, and then incubated with the primary antibodies against E2F1 (Santa Cruz Biotechnology, sc-251, 1:250) overnight at 4ºC. Cells were washed with 0.1% Triton-X100/PBS and incubated with Alexa Fluor®-conjugated secondary antibodies for 1 h at RT, and the nuclei were stained with the DAPI dye. Images were taken with the Thermo Scientific™ Invitrogen™ EVOS™ FL Auto 2 Imaging System.

Flow Cytometry

To determine the transfection efficiency of cultured CEC, we used a plasmid coding for GFP (pCAG-GFP). Cells were trypsinized and collected by centrifugation at 200 g for 5 m at RT. The pellets were resuspended in PBS for washing and then fixed in 4% paraformaldehyde for 10 m at RT. Cells were then washed and the pellets were resuspended in 200 µL PBS buffer.

Data were acquired on a Beckman Coulter CytoFLEX LX cytometer using CytExpert v2.5 (Beckman Coulter) and analyzed using FlowJo (Treestar Inc.). Cells were first gated (SSC-A vs FSC-A), and then singlets were gated (FSC-H vs FSC-W). Fluorescence from GFP-expressing cells was measured using the GFP488_525-40-A channel.

Relative gene expression

Sequences of primers:

| Gene name | Specie | Fw/Rv | Primer sequence |
| --- | --- | --- | --- |
| *Cdh5* | Mouse | Fw | 5’-GAACGAGGACAGCAACTTCACC-3’ |
|  |  | Rv | 5’-GTTAGCGTGCTGGTTCCAGTCA-3’ |
| *Aqp4* | Mouse | Fw | 5’-AGCCAGCATGAATCCAGCTCGA-3’ |
|  |  | Rv | 5’-TCATAAAGGGCACCTGCCAGCA-3’ |
| *Cspg4* | Mouse | Fw | 5’-GAGGTCTTGGTGAACTTCACCC-3’ |
|  |  | Rv | 5’-GACAGTAGGAGACCGATGGTGT-3’ |
| *Mog* | Mouse | Fw | 5’-GATGAAGGAGGCTACACCTGCT-3’ |
|  |  | Rv | 5’-CGTAGGCACAAGTGCGATGAGA-3’ |
| *Nefl* | Mouse | Fw | 5’-GCCTTGGACATCGAGATTGCAG-3’ |
|  |  | Rv | 5’-CAAGCCACTGTAAGCAGAACGG-3’ |

Immunohistochemistry-immunofluorescence

For CD31 and GFP staining, brain slices were incubated with a blocking buffer (5% BSA and 0.1% Triton-X100 in PBS) for 1 h at RT. Then, slices were incubated with anti-CD31 (Abcam, #ab28364, 1:250) and anti-GFP (Abcam, #ab290, 1:250) dissolved in a blocking buffer overnight at 4ºC. Slices were then washed with 0.1% Triton-X100 twice for 5 m at RT and incubated with Alexa-conjugated secondary antibodies for 1 h at RT. Slices were washed twice and then mounted with Fluoroshield mounting medium with DAPI.

An investigator blinded to the experimental groups took the images with a Leica DMi8 microscope system.

**Supplementary Figure Legends**

**Supplementary Figure 1. Characterization of cerebral microvessel isolated from aged mice.** A) Representative image of a cerebral microvessel isolated from a 20-m/o male mice and stained with antibodies against the endothelial marker CD31 (red) and the nuclear DAPI dye (blue). Scale bar, 50 µm. *B)* Gene expression of *Cdh5* (endothelial), *Aqp4* (astrocyte), *Cspg4* (oligodendrocyte precursor cells), *Mog* (mature oligodendrocyte), *Nefl* (neuron) relative to *Gapdh* expression in the cerebral microvessel fraction of 3 aged mice.

**Supplementary Figure 2. Transfection efficiency in aged mouse-derived CEC.** *A)* Cultured CEC isolated and cultured from 20-m/o male mice were transfected with the plasmid pCAG-GFP and observed with a microscope using the brightfield and the GFP channels. Scale bar, 100 µm. *B)* Scatter plots of flow cytometry from aged mouse-derived CEC transfected with pCAG-GFP (GFP Ag CEC, green) . We used CEC derived from young (Control Yg CEC, blue) and aged mice (Control Ag CEC, red) and transfected them with an empty plasmid (pCMV-HA) as controls. Note that the percentage of GFP-positive cells resulting from subtracting Control Ag CEC from GFP Ag CEC is about 35%. *C)* Aged mouse-derived CEC were co-transfected with the plasmids pCAG-GFP and pCMV-HA-E2F1. Cells were fixed and stained with antibodies against E2F1 (red) and with the nuclear DAPI dye (blue). The green fluorescent signal is from the transfection with the pCAG-GFP plasmid. Scale bar, 10 µm.


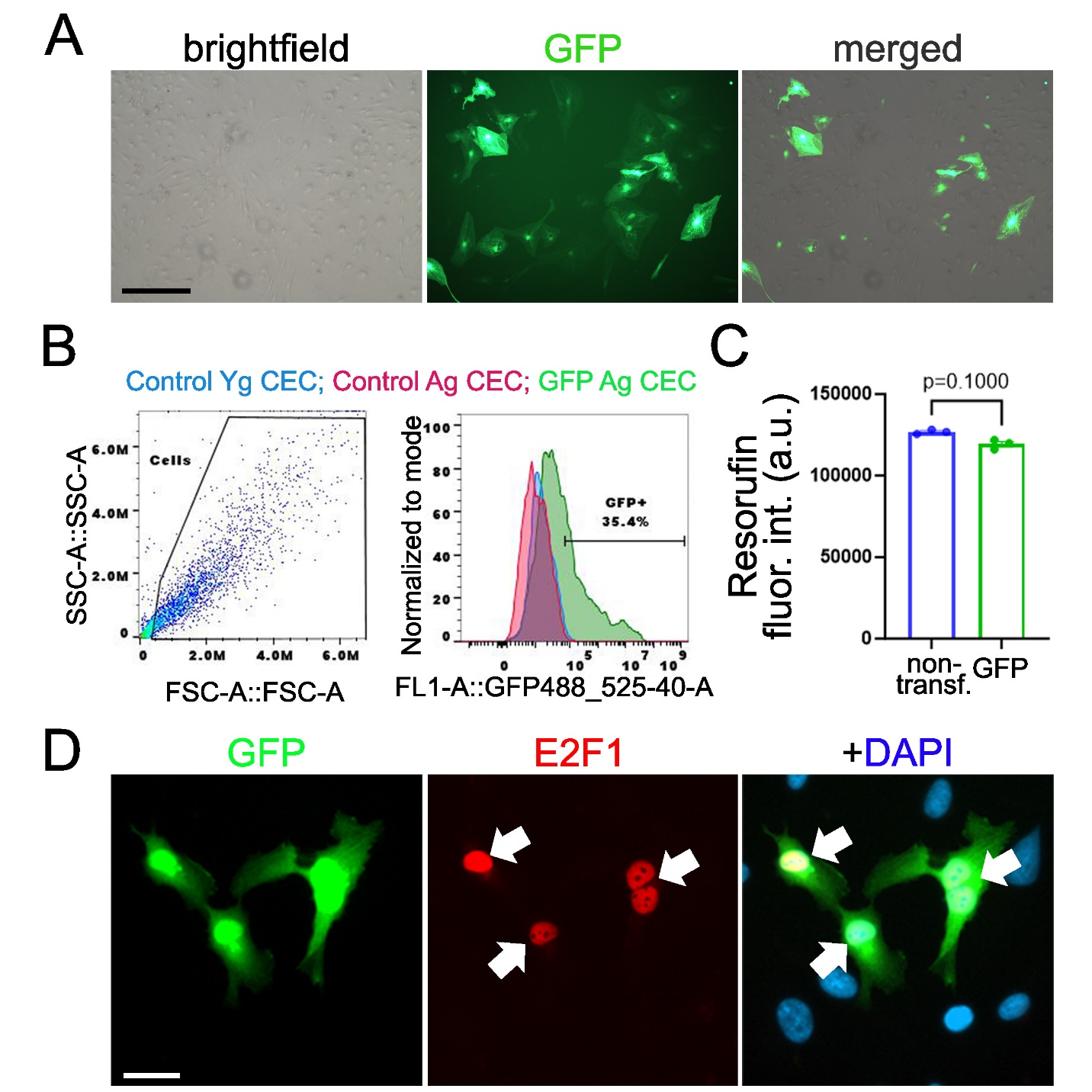


**Supplementary Figure 3.** **The AAV(BR1) vector specifically targets the brain vasculature of aged mice.** A) Representative images of the hippocampus (top panel) and cortex (bottom panel) of a 20-m/o male mouse injected with AAV(BR1)-CAG-mE2F1-T2A-GFP 2 months before euthanasia. Brain samples were stained with anti-CD31 (red) and with the nuclear DAPI dye (blue). Scale bar, 100 µm. Note that the GFP signal (green) co-localizes with CD31-positive vessels. B) Representative images of GFP-positive neurons in the brain of a 20-m/o male mouse injected with AAV(BR1)-CAG-T2A-GFP and later stained with anti-CD31 (red) with the nuclear DAPI dye (blue). Scale bar, 25 µm.

**Supplementary Figure 4. Optimization of the concentration of a derivative itaconate to prevent H_2_O_2_-induced cell death.** Quantification of the fluorescence intensity Cell Titer-Blue (Resorufin) in CEC pre-treated with different concentrations of 4-OI (10, 25, 50 µM) or a vehicle, for 1 h, and then treated with H_2_O_2_ (500 µM), or a vehicle for 2 h. Then, the medium was replaced with complete endothelial medium for 24 h. The CellTiter-Blue reagent was added, and 4h later, the fluorescence was measured. Data are mean ± SEM pooled from 8 replicates from three mice. Two-way ANOVA test, Tukey’s multiple comparisons test.
